# Molecular Changes Associated with Inflammation and Reproduction in Cadmium-Induced Testicular Toxicity: Mitigating Effect of *Lactobacillus plantarum*

**DOI:** 10.1007/s12011-025-04601-5

**Published:** 2025-04-04

**Authors:** Duygu Kizir, Emine Toraman, Melike Karaman

**Affiliations:** Department of Molecular Biology and Genetics, Faculty of Science, Atatürk University, Erzurum, 25240 Turkey

**Keywords:** Heavy metal, *Lactobacillus plantarum*, Testicular toxicity

## Abstract

Cadmium (Cd) is a dangerous heavy metal that causes toxicity in humans and animals. Various protective agents are being investigated to ameliorate the toxic effect of Cd. Lactic acid bacteria are promising as a protective agent because of their ability to remove heavy metals from contaminated products. The aim of this study was to evaluate the effects of *Lactobacillus plantarum* on biomarkers associated with reproduction and inflammation in the testes of rats exposed to Cd. For the application, rats were randomly divided into four groups: Control group, Cd group, *L. plantarum* group, and combined group (Cd + *L. plantarum*). At the end of 21 days of oral administration, testicular tissues of the rats were removed and mRNA expression levels of genes associated with reproduction (*Dazl*, *Amh*, and *Ddx4*) and inflammation (*Tnf-α*, *Il-6*, *Cox-2*, *Inos*, *Foxo1*, *Foxo3*, and *Nfkb*) were determined. On the other hand, the amounts of NOS2/iNOS, 8-ohdg, and Tnfα were analyzed in the tissues. The mtDNA copy number was also investigated. Cd caused an increase in the expression level of inflammation-related genes, except *Nfkb*, and a decrease in the expression level of reproduction-related genes. It also increased the levels of TNF-α, iNOS, and 8-OHdG biomarkers and mtDNA copy number. However, *L. plantarum* treatment did not cause a significant change in these parameters. Moreover, *L. plantarum* exposure in combination with Cd attenuated the observed Cd-mediated molecular changes in testicular tissue. In conclusion, the findings suggest that *L. plantarum* administration may be beneficial against Cd-induced testicular toxicity and may be considered as a biological agent in the development of protective strategies against environmental pollutants with further studies.

## Introduction

Cadmium (Cd) is a transition metal that is a hazardous substance due to its toxic effects. It is found as a pollutant in the environment as a result of agricultural and industrial activities such as copper and nickel smelting and refining, fossil fuel combustion, and the use of phosphate fertilizers [1]. Additionally, it also contaminates the environment as a byproduct of zinc production [2]. Exposure to Cd can occur through ingestion of contaminated food and beverages, smoking, and occupational exposure. Cd exposure causes toxicity in humans and animals and affects the function of organs such as kidneys, liver, lungs, and testes [1, 3]. Cd toxicity has been attributed to a number of mechanisms, including metal dyshomeostasis, oxidative stress, inflammation, and chromosomal instability. It has also been reported to disrupt intestinal physiology [4]. Anthropogenic activities increase the amount of Cd in the environment and, as a consequence, lead to Cd accumulation in plants and animals along the food chain [5]. Due to its increasing importance as an environmental pollutant, there is a need for studies on useful solutions to prevent or remedy the harmful effects of Cd on the environment and human health.

The use of microbial techniques to remove the polluting effect of heavy metals is increasing. Microorganisms fulfill this function through adsorption and bioaccumulation. Lactic acid bacteria (LAB), in particular, are considered suitable sorbents due to their beneficial effects and the fact that they are considered safe [6]. LAB was used to perform microbial remediation of heavy metals from aqueous medium [7]. Heavy metals and toxins in food can be bio-decontaminated by strains like *Bacillus*, *Bifidobacterium*, *Lactobacillus*, *Lactococcus*, *Enterococcus*, *Pediococcus*, *Propionibacterium*, *Streptococcus*, and *Saccharomyces cerevisiae* [8]. In addition, the ameliorative effects of LAB against heavy metal toxicity due to their oxidative stress tolerance and antioxidant capacity have also been investigated [9–11]. In lead (Pb)-treated mice, *Lactiplantibacillus plantarum* treatment was reported to significantly reduce Pb accumulation by inducing Pb excretion and increase antioxidant capacity [12]. On the other hand, in mice in which a high-fat diet and Pb caused dysbiosis of the gut microbiota and impaired spermatogenesis, probiotic supplementation ameliorated these adverse effects [13]. Therefore, the usability of LAB in the search for protective agents against heavy metal toxicity comes to the fore.

There is evidence that Cd causes toxicity and decreased sperm quality in the testicular tissue of rats [14, 15]. Natural compounds such as theaflavins, α-tocopherol, naringenin, curcumin, and quercetin have been shown to have protective potential in the elimination of Cd damage in testicular tissue [14, 16–19]. The Nrf2/ARE signaling pathway has been reported to be involved in the induction of Cd-induced oxidative stress and apoptosis of testicular germ cells, but the exact mechanisms remain unclear [20]. In this study, it has planned to contribute to the elucidation of the processes in Cd-induced testicular toxicity and to examine the effect of probiotic bacteria against this toxicity. For this purpose, the effect of *L. plantarum* against Cd-induced testicular toxicity was investigated in terms of changes in biomarkers related to reproduction and inflammation.

## Material and Methods

### Bacterial Culture

*L. plantarum* ACC54 strain was cultured on de Man-Rogosa-Sharpe Agar (MRS, Merck) at 37 °C for 48 h. The colonies were then suspended in PBS solution at a concentration of approximately 10^9^ CFU/mL and used in the applications [21, 22]. The bacterial strain is a generous gift of Prof. Dr. Bülent Çetin.

### Conditions of Animal Care and Experimental Groups

The rats (*Sprague–Dawley* male, 180 ± 20 g, n: 20) used in the study were obtained from Atatürk University Medical Experimental Research and Application Center (ATADEM). The treatments were performed at ATADEM in accordance with the national guidelines for the use and care of experimental animals. Animals were housed in steel cages in an environment with a temperature of 22 ± 2 °C, 55 ± 10% humidity, and 12 h light-12 h dark cycle. Rats were allowed access ad libitum to feed and water throughout the study. This study was approved by Atatürk University Animal Experiments Local Ethics Committee (No: 2024/12–263).

Four groups (n: 5) of twenty rats were randomly selected.

- Control group: Normal saline (0.9%) was given daily by oral gavage for 21 days.
- Cd group: CdCl_2_ (5 mg/kg bw/day) was administered by oral gavage for 21 days.
- *L. plantarum* group: Bacterial solution (1 × 10^9^ CFU/day) was administered by oral gavage for 21 days.
- Cd + *L. plantarum* group: The treatments in the 2nd and 3rd groups were performed together for 21 days.

The CdCl_2_ concentration and the number of bacteria used in the treatment were determined with reference to the literature [23, 24]. At the end of the treatments, rats were sacrificed by cervical dislocation under anesthesia. The testicular tissues were frozen in liquid nitrogen and stored at − 80 °C for analysis.

### Molecular Analysis

#### RNA Isolation and cDNA Synthesis from Testicular Tissue

RNA isolation from testicular tissues of all groups was performed using RNA extraction kit (Invitrogen) according to the manufacturer’s instructions. The concentration and quality of the isolated RNAs were determined on a nanodrop device (Thermo Scientific™ Multiskan™). These RNAs were then converted into cDNA using cDNA synthesis kit (A.B.T™) according to the manufacturer’s instructions. cDNAs were stored at − 20 °C for use in real-time PCR.

#### Quantitative Gene Expression Analysis (Real-Time PCR)

Real-time PCR to analyze the expression of genes associated with inflammation (*Tnf-α*, *Il-6*, *Cox-2*, *Inos*, *Foxo1*, *Foxo3*, and *Nfkb*) and reproduction (*Dazl*, *Amh*, and *Ddx4*) in testicular tissues was performed on a Rotor-Gene Q instrument (Qiagen) using SYBR Green master mix (Biorad). The PCR reaction was prepared with 12.5 µl of master mix, 0.25 µl of each primer pair, and 2.5 µl of cDNA for a total of 25 µl. PCR conditions are as follows: 1 cycle at 50 °C for 2 min and 95 °C for 10 min, 45 cycles at 95 °C for 10 s, and extension at 60 °C for 1 min. The sequences of the primers employed in the experiment are provided in Table 1. The *C*_T_ values obtained were analyzed using the ΔΔC_T_ method [25].

**Table 1:** Sequences of the primers utilized in the experiment

Genes*	Forward primer sequences (5′ → 3′)	Reverse primer sequences (5′ → 3′)	Accession number
*Tnf-α*	AGGAGGGAGAACAGCAACTC	TGTATGAGAGGGACGGAACC	NM_012675.3
*Il-6*	AGTTGCCTTCTTGGGACTGA	TACTGGTCTGTTGTGGGTGG	NM_012589.2
*Cox-2*	TTCGGGAGCACAACAGAGTG	TGGAACAGTCGCTCGTCATC	L25925.1
*Inos*	GGATATCTTCGGTGCGGTCTT	CTGTAACTCTTCTGGGTGTCAGA	U03699.1
*Foxo1*	ACCGTATCTGTGTGTGTGTGTG	ACAGCCAAGTCCATCAAGAC	NM_001191846.3
*Foxo3*	ACTGAGGAAAGGGGAAATGG	TGCTGGGTTAGGAAGATGGC	NM_001106395.1
*Nfkb*	GATCTCGACCTCCACCGGAT	TTCCCAGAGTTTCAGACGCC	NM_012611.3
*Dazl*	GTCTTCATCAGCAACCACCAG	CATCCATCCTAACATCAATTCCAC	NM_001413924.1
*Amh*	AATACCAGGGGCCTCATCT	TCAGTACTGCAGACTCCGAA	NM_012902.2
*Ddx4*	CAGCATTCCCATTGTGTTAGC	GGCAGTTATTCCATCCCTCATC	NM_001077647.2
*Gapdh*	TGGACCTCATGGCCTACATG	AGGGAGATGCTCAGTGTTGG	NM_017008.4

*Tnf-α tumor necrosis factor-α, *Il-6* interleukin-6, *Cox-2* cyclooxygenase-2, *Inos* inducible nitric oxide synthase, *Foxo1* Forkhead box O1, *Foxo3* Forkhead box O3, *Nfkb* nuclear factor kappa B, *Dazl* deleted in azoospermia 1, *Amh* anti-Müllerian hormone, *Ddx4* DEAD-box polypeptide 4, *Gapdh* glyceraldehyde-3-phosphate dehydrogenase

#### Determination of Mitochondrial DNA (mtDNA) Copy Number

Genomic DNA was obtained from testicular tissues using DNA isolation kit (Ecopure) according to the manufacturer’s instructions. The mtDNA copy number was carried out by real-time PCR analysis using SYBR Green master mix (Biorad) following the producer’s directions. The forward and reverse primer sequences for nuclear gene (18S rRNA) were 5′-GAC TCA ACA CGG GAA ACC TC-3′ and 5′-TAA CCA GAC AAA TCG CTC CA-3′, respectively. The forward and reverse primer sequences for mtDNA (D-loop region) were 5′-AGG CAT CTG GTT CTT ACT TCA G-3′ and R 5′-TGA CGG CTA TGT TGA GGA AG-3′, respectively. mtDNA copy number was calculated with the formula 2^(nucDNA C_T_—mtDNA C_T_) [26].

### Biochemical Analysis

Testicular tissues were homogenized in 50 mM phosphate buffer (pH: 7.4) containing 1 mM EDTA and 1 mM DTT in a 1:10 ratio in a TissueLyser LT device (Qiagen) set at a frequency of 50 (1/s) for 1 min. Homogenates were centrifuged at 10,000 rpm for 30 min at 4 °C. The supernatants were collected and used for biochemical analysis. Total protein content of testicular tissues of all groups was determined spectrophotometrically at 595 nm using the Bradford method [27]. Bovine serum albumin (Sigma) was used in the preparation of the standard graph.

The inducible nitric oxide synthase (iNOS), 8-hydroxy-2′-deoxyguanosine (8-OHdG), and tumor necrosis factor-α (Tnf-α) levels in the testicular tissues of each group were determined using NOS2/iNOS ELISA kit (Reed Biotech Ltd), 8-ohdg ELISA kit (ELK Biotechnology Co., Ltd), and Tnf-α ELISA kit (Shanghai YL Biotech Co., Ltd) according to the manufacturer’s instructions, respectively. Measurements were performed on a Microplate Photometer (Thermo Scientific™ Multiskan™).

### Statistical Analysis

Data from the experiments are presented as mean ± standard deviation (SD). GraphPad Prism 8.0 (GraphPad Software, San Diego, CA) was used to evaluate the results. Statistical significance between groups was analyzed using one-way ANOVA and Tukey’s post-hoc test. Statistical significance was accepted as *p* < 0.05.

## Results

### Changes in the Expression Level of Inflammation-Related Genes

**Fig. 1:**
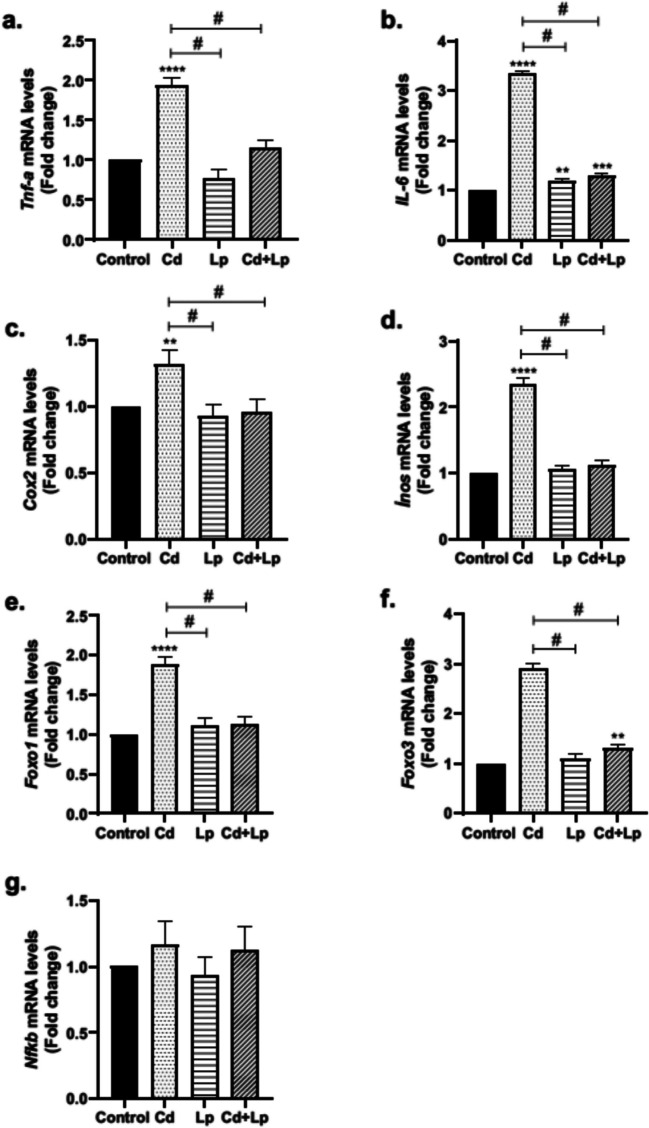
Differences in the relative expression levels of *Tnf-α*, *Il-6*, *Cox-2*, *Inos*, *Foxo1*, *Foxo3*, and *Nfkb* genes associated with inflammation in testicular tissue after Cd and/or *L. plantarum* exposure (**a**-**g**). Cd, Cd exposure; Lp, *L. plantarum* exposure; Cd + Lp, co-exposure of Cd and *L. plantarum*. Measures are provided as mean + SD. Asterisk (*) refers to statistical significance compared to control (*p < 0.05, **p < 0.01, ***p < 0.001); number sign (#) refers to statistical significance between groups (# *p* < 0.05)

*Tnf-α*, *Il-6*, *Cox-2*, *Inos*, *Foxo1*, *Foxo3*, and *Nfkb* genes are biomarkers involved in inflammation. The differences observed in the relative expression levels of these genes in rat testicular tissues after Cd administration, *L. plantarum* treatment, and co-exposure of *L. plantarum* with Cd are illustrated in Fig. 1. Cd exposure produced a statistically significant elevation in the gene expression level of all genes in the tissues except the *Nfkb* gene. However, *L. plantarum* intake did not cause a statistically significant change in the genes except *Il-6*. On the other hand, *L. plantarum* treatment with Cd reduced the Cd-induced increase in gene expression levels closer to the values of the control group.

### Changes in the Expression Level of Reproduction-Related Genes

**Fig. 2:**
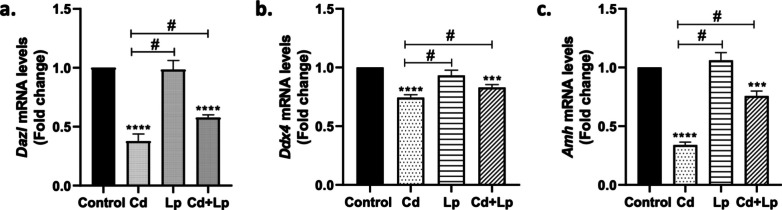
Changes in relative mRNA levels of reproduction-related genes (*Dazl*, *Ddx4* and *Amh*) in testicular tissue after Cd and/or *L. plantarum* exposure (**a**-**c**). Cd, Cd exposure; Lp, *L. plantarum* exposure; Cd + Lp, co-exposure of Cd and *L. plantarum*. Asterisk (*) refers to statistical significance compared to control (*p < 0.05, **p < 0.01, ***p < 0.001); number sign (#) refers to statistical significance between groups (# *p* < 0.05)

The *Dazl*, *Ddx4*, and *Amh* genes are associated with gametogenesis and sperm generation [28]. Relative mRNA expression levels of these genes associated with reproduction were evaluated in the testicular tissue of the Cd group, *L. plantarum* group, and the combined group in which Cd and *L. plantarum* were administered together. Alterations in expression levels are reported in Fig. 2. Cd exposure decreased mRNA levels of the reproduction-related genes. Co-exposure of Cd and *L. plantarum* did not affect Cd-induced changes. On the other hand, *L. plantarum* treatment did not cause a statistically significant change in mRNA levels.

### Changes in TNF-α, iNOS, and 8-OHdG Biomarkers

**Fig. 3:**
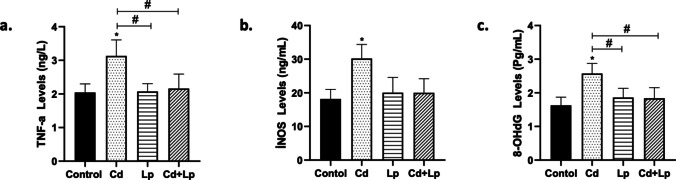
Changes in TNF-α levels, iNOS activity, and 8-OHdG levels in testicular tissue after Cd and/or *L. plantarum* exposure (**a**-**c**). Cd, Cd exposure; Lp, *L. plantarum* exposure; Cd + Lp, co-exposure of Cd and *L. plantarum*. Asterisk (*) refers to statistical significance compared to control (*p < 0.05, **p < 0.01, ***p < 0.001); number sign (#) refers to statistical significance between groups (# *p* < 0.05)

TNF-α, iNOS, and 8-OHdG are interrelated biomarkers analyzed as indicators of inflammation, oxidative stress, and cellular damage [29]. The effects of Cd exposure and co-exposure of Cd with *L. plantarum* on TNF-α levels, iNOS enzyme activity, and 8-OHdG levels in testicular tissue were observed (Fig. 3). The results showed that Cd exposure resulted in a statistically significant rise in TNF-α and 8-OHdG levels and iNOS enzyme activity in tissues. In contrast, co-exposure of Cd with *L. plantarum* diminished this Cd-induced rise.

### Changes in Mitochondrial DNA Copy Number

**Fig. 4:**
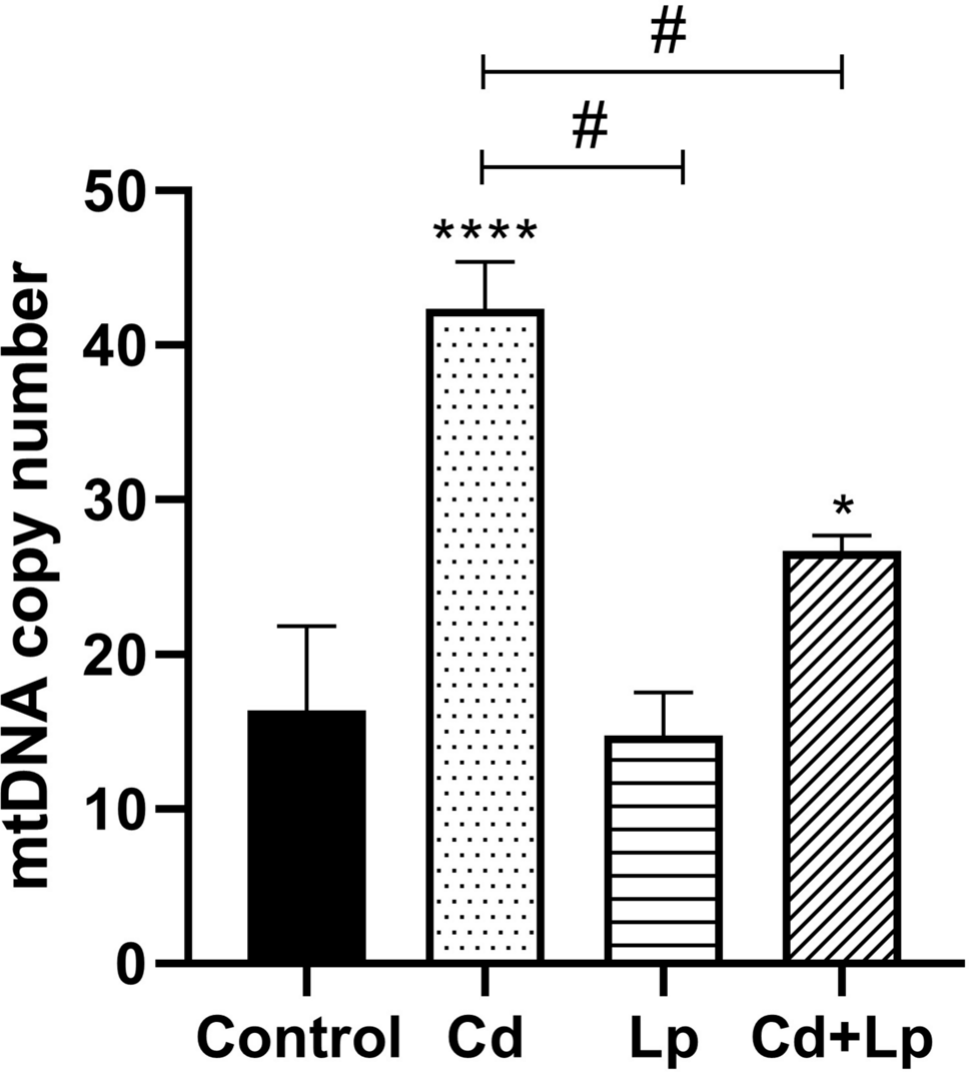
Effect of Cd and/or *L. plantarum* exposure on mtDNA copy number in testicular tissue. Cd, Cd exposure; Lp, *L. plantarum* exposure; Cd + Lp, co-exposure of Cd and *L. plantarum*. Asterisk (*) refers to statistical significance compared to control (*p < 0.05, **p < 0.01, ***p < 0.001); number sign (#) refers to statistical significance between groups (# *p* < 0.05)

Mitochondrial DNA (mtDNA) is vulnerable to environmental stress as it lacks histone protection and an efficient DNA repair system and is considered a biomarker to understand the level of stress exposure [30]. Changes in mtDNA copy number in testicular tissues after Cd treatment and co-treatment of Cd and *L. plantarum* are depicted in Fig. 4. Cd exposure enhanced mtDNA copy number in tissues. Co-exposure of Cd and *L. plantarum* caused a statistically significant decrease in the Cd-induced increase.

## Discussion

Cd is transmitted to the environment as a result of agricultural and industrial activities and is recognized as a polluting toxic agent [1]. Cd exposure causes dysfunction in organs as well as hepatoxicity, nephrotoxicity, neurotoxicity, and testicular toxicity [31–34]. It also increases the risk of cancer [2]. Therefore, investigations to search for protective agents against Cd toxicity are important. In studies conducted for this purpose, compounds such as quercetin, resveratrol, and vitamin E, which are naturally found in plants against Cd toxicity, as well as lactic acid bacteria are presented as a good option to prevent toxic effects [35–38]. In the present study, we evaluated the changes in the testes of rats exposed to Cd toxicity and the effect of *L. plantarum*, a lactic acid bacterium, on these changes in terms of biomarkers associated with reproduction and inflammation.

Cd toxicity in testes has been reported to exhibit oxidative, apoptotic, steroidogenic, and spermatotoxic effects. Cd-induced testicular toxicity was investigated in terms of steroidogenic acute regulatory protein (StAR) and 17-hydroxy steroid dehydrogenase (17-β HSD) gene expression, phosphatase level, and changes in testicular function and the effects of *Lactobacillus* and *Acidobacillus* supplementation against these changes were investigated. Bacteria were found to modulate Cd-induced decreases in these parameters [20]. However, the effect of Cd was not evaluated in terms of changes in reproduction-related *Dazl*, *Ddx4*, and *Amh* genes. Changes observed in the genes *Dazl* and *Ddx4*, germ cell markers, and *Amh*, a marker for Sertoli cells, in testicular tissues have been associated with fertility disorders and infertility [28]. In the present study, we investigated the expression levels of these genes associated with reproduction in Cd-induced testicular toxicity. The results from the study revealed that Cd causes a decrease in the mRNA level of these genes in testicular tissue. Nevertheless, exposure to *L. plantarum* could not alter this reduction. This suggests that bacteria may act on different pathways against Cd toxicity. Therefore, it shows that *L. plantarum* may have ameliorative potential in terms of different pathways against testicular toxicity, although it does not affect reproduction-related genes.

Oxidative stress, which plays an important role in Cd toxicity, triggers inflammation. Therefore, while investigating the effect of protective agents against Cd toxicity, inflammation-related parameters are evaluated in addition to oxidative stress. On the other hand, it is also known that cellular signaling pathways including the NF-κB pathway are involved in this toxicity through direct or indirect mechanisms. Cd may initiate inflammatory responses by inducing cytokines such as TNF-α, IL-1β, and IL-6 through activation of the NF-κB pathway [39]. Moreover, it also alters the activity of inflammation-related enzymes Cox-2 and iNOS [40]. Therefore, in the present study, the effect of *L. plantarum* on the prevention/mitigation of Cd-induced changes in inflammation-related mediators was examined in testicular tissue of rats. The results showed that *L. plantarum* reduced Cd-induced increased mRNA expression in inflammation-related genes, TNF-α level, and iNOS activity. Forkhead box transcription factors (FOXO) are involved in several physiological processes including cytokine expression, inflammation, and resistance to oxidative stress. FOXO1 and FOXO3 members are expressed in almost all tissues [41]. Therefore, Cd-induced changes in testicular tissue were analyzed through these members. Cd exposure caused an increase in *Foxo1* and *Foxo3* mRNA levels, while the addition of *L. plantarum* attenuated this increase. Cd-induced increases in inflammation-related genes may also be interrelated because *Foxo1* is strongly activated by TNF-α. On the other hand, cooperation between NF-κB and *Foxo1* has been reported to increase inflammation. Furthermore, NF-κB regulates genes for enzymes such as COX-2 and iNOS and cytokines such as IL-6, IL-8, and TNF [41, 42].

Since Cd-induced oxidative stress causes an increase in reactive oxygen species (ROS), ROS production can be evaluated in terms of mtDNA copy number and 8-OHdG. In a study, the use of a commercial probiotic Protexin® and its nanoformulation against Cd toxicity was found to significantly reverse Cd-induced changes in malondialdehyde (MDA) and 8-hydroxydeoxyguanosine (8-OHdG) levels in serum of male Wistar rat [43]. Our results showed that Cd caused an increase in mtDNA copy number and 8-OHdG levels in testicular tissue. On the other hand, exposure to *L. plantarum* together with Cd attenuated this increase. mtDNA copy number is a biomarker of mitochondrial genome alterations, mitochondrial dysfunction, and excessive generation of reactive oxygen species (ROS) [44]. Increased ROS leads to an increase in mtDNA copy number, which is indicative of oxidative damage [45].

All findings suggest that bacteria may act in different pathways against Cd toxicity. Therefore, it suggests that *L. plantarum* may have ameliorative potential in terms of different pathways against testicular toxicity, although it does not affect reproduction-related genes. However, further studies are needed to fully characterize Cd-induced testicular toxicity and the ameliorative role of *L. plantarum* in Cd-induced testicular toxicity.

## Conclusion

This study revealed that Cd exposure decreased the levels of reproduction-related genes *Dazl*, *Amh*, and *Ddx4* in testicular tissue and that *L. plantarum* was effective in Cd-induced testicular toxicity via inflammation. This bacterium attenuated the Cd-induced increase in mRNA levels of inflammation-related genes, mtDNA copy number, and iNOS and 8-OHdG levels. *L. plantarum* administration may be a good approach to counteract the adverse effects of Cd and possibly other heavy metals.

## Data Availability

No datasets were generated or analysed during the current study.
